# Use of Ultrasound for Navigating the Internal Carotid Artery in Revision Endoscopic Endonasal Skull Base Surgery

**DOI:** 10.7759/cureus.13547

**Published:** 2021-02-25

**Authors:** Jonathan P Giurintano, Jose Gurrola, Philip V Theodosopoulos, Ivan H El-Sayed

**Affiliations:** 1 Otolaryngology - Head and Neck Surgery, MedStar Washington Hospital Center, Washington, USA; 2 Otolaryngology - Head and Neck Surgery, University of California, San Francisco, USA; 3 Neurological Surgery, University of California, San Francisco, USA

**Keywords:** skull base, ultrasound, endoscopic, chordoma

## Abstract

While the use of image-guided navigation is an excellent adjunct to the use of anatomical landmarks, dynamic changes that may occur in the position of critical structures are not accounted for during and after tumor resection. Unlike navigation, Doppler ultrasonography provides real-time imaging of the anterior skull base and can be used to accurately identify the location of vital structures during skull base surgery.

A 56-year-old female initially presented with new onset left eye visual deficits. She previously underwent sublabial transsphenoidal subtotal resection of the tumor, confirmed as clival chordoma. She subsequently presented to our institution. She was treated with an expanded endonasal resection of the remaining chordoma followed by CyberKnife radiosurgery. Two years later, surveillance imaging identified tumor recurrence within the right clivus posterior to the carotid artery. Intraoperatively, in the previously operated irradiated skull base, the normal bony architecture of the sella was absent, resulting in the inability to distinguish the anterior genu of the internal carotid artery (ICA) from the adjacent tumor. Using Doppler ultrasonography, the course of the ICA was imaged in real time, allowing for safe, gross total tumor resection.

In the setting of prior operation, radiation, or extensive disease, the normal bony architecture of the sella may be disrupted, placing the cavernous ICA at risk. We report what we believe is the first use of intraoperative ultrasound during the endoscopic endonasal approach in the setting of a previously operated, radiated sella.

## Introduction

Endoscopic surgery for lesions of the pituitary gland is now commonplace. In the setting of prior operation, radiation, or extensive disease, the normal bony architecture of the sella may be disrupted, disorienting the surgeon during an endoscopic approach to the anterior skull base, and possibly placing the cavernous internal carotid artery (ICA) at risk. Revision transsphenoidal surgery was traditionally associated with lower rates of complete resection, higher complication rates, and worse outcome compared with the primary operation, largely due to the presence of mucosal adhesions, altered anatomy, and increased difficulty in visualization. Increasing experience with endoscopic endonasal surgery has allowed more complex resections with lower rates of major complication, and revision endonasal surgery has shown to have similar resection rates and similar complication rates to primary surgery [1,2].

One of the major advances in endoscopic skull base surgery has been the widespread use of image-guided navigation systems to assist in the identification of critical anatomical structures. While generally an excellent adjunct to the use of anatomical landmarks, image-guided navigation is limited by its reliance on pre-operative, pre-surgical anatomy. Dynamic changes that may occur in the position of critical structures are not accounted for during and after tumor resection. Unlike navigation, Doppler ultrasonography provides real-time imaging of the anterior skull base and can be used to accurately identify the location of vital structures during surgery. With the advent of advanced technology, the feasibility of using Doppler ultrasonography during endoscopic endonasal skull base surgery has previously been demonstrated in the primary resection of a tuberculum sella meningioma with positive results [3]. To our knowledge, the endoscopic ultrasound has not been used in the setting of revision transsphenoidal surgery. We report our experience using endoscopic Doppler ultrasonography in a particularly difficult case to highlight the potential application of ultrasound imaging to endoscopic skull base surgery. This article was previously presented as a poster at the 28th Annual North American Skull Base Society Meeting in Coronado, CA, on February 15, 2018.

## Case presentation

A 56-year-old female initially presented to an outside hospital with a new onset of left eye visual deficits while visiting her daughter. Imaging revealed a lesion in the clivus extending into the sella, and she underwent a sublabial transsphenoidal subtotal resection of the tumor, with post-operative pathology confirmed to be clival chordoma. She subsequently presented to our institution. After evaluation, our minimally invasive skull base team performed an expanded endonasal resection of the remaining chordoma within the left clivus along the petro-clival synchondrosis and posterior to the ICA, extending into the inferior cavernous sinus. Gross total tumor resection was performed, with a high-flow cerebrospinal fluid (CSF) leakage present at the completion of the resection. The resultant skull base defect was reconstructed with an abdominal fat graft, sutured to two layers of collagen matrix and nasoseptal flap. After an uncomplicated post-operative course, she received 4,000 cGy of CyberKnife radiosurgery in five fractions for residual disease and remained symptom-free for two years.

**Figure 1:**
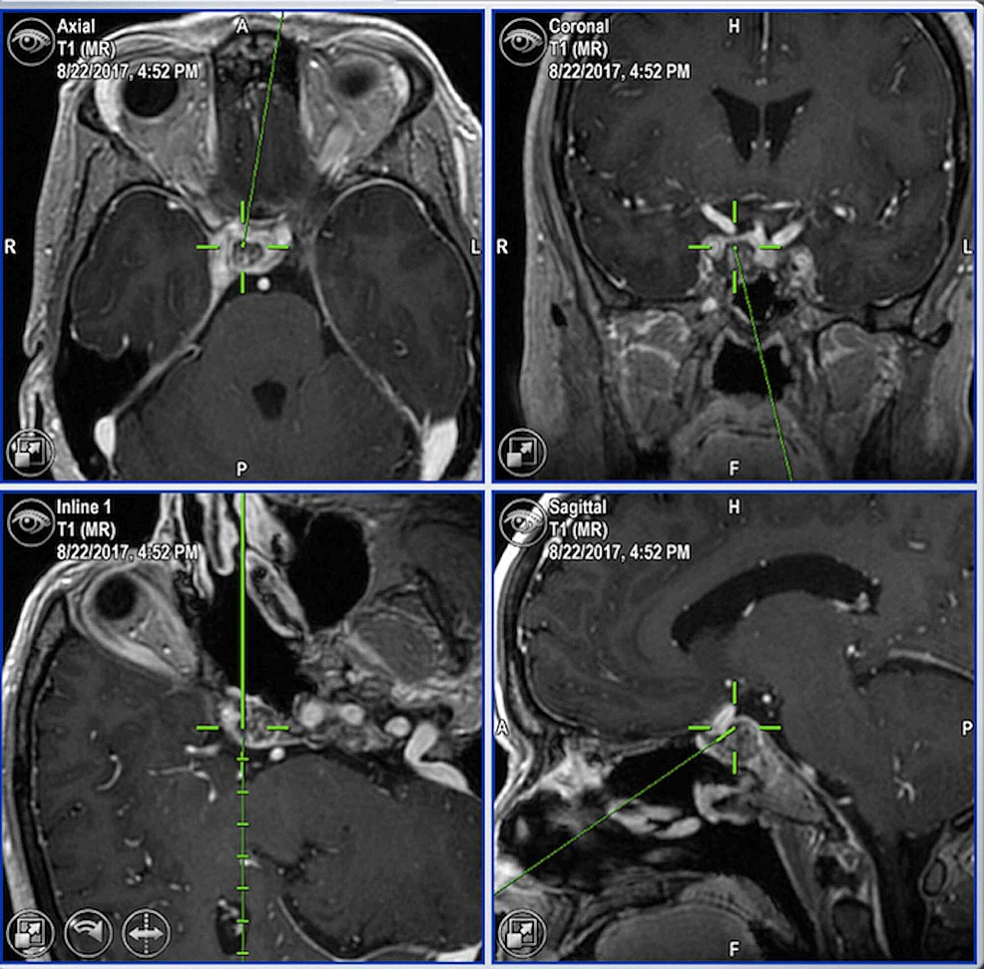
Neuronavigation demonstrating right retrocarotid clival tumor recurrence

**Figure 2:**
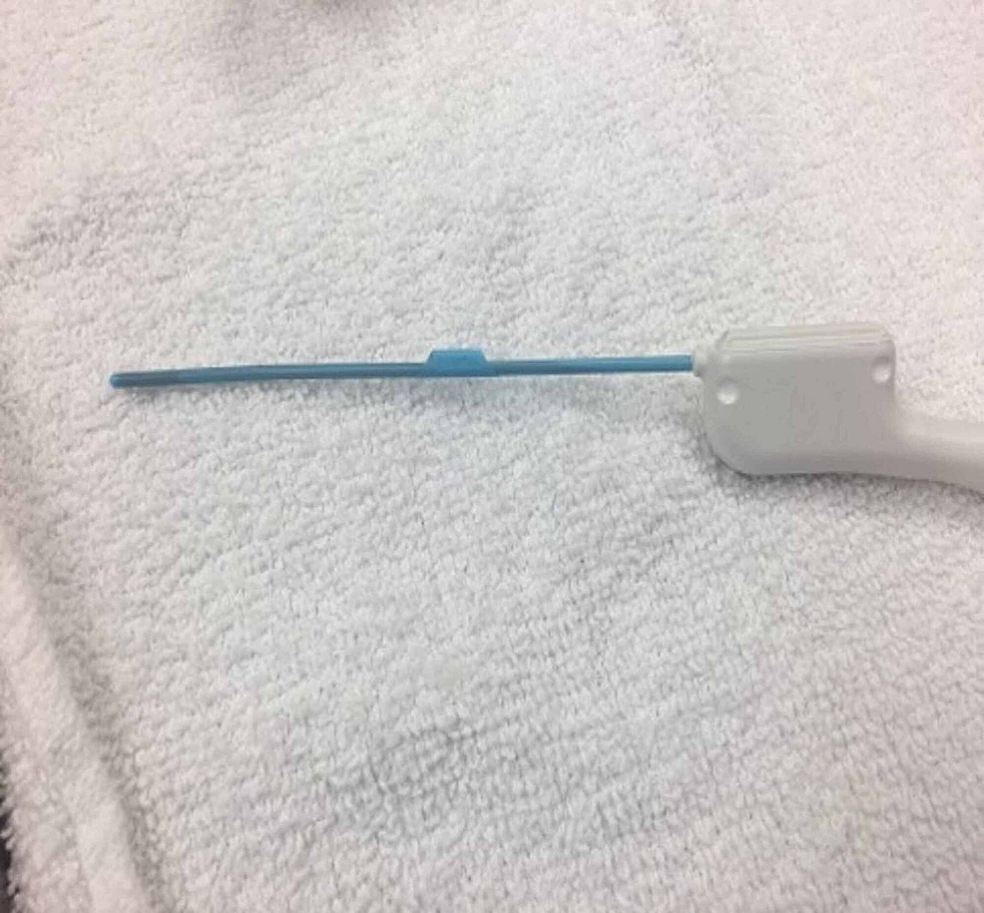
Hitachi pituitary transducer

**Figure 3:**
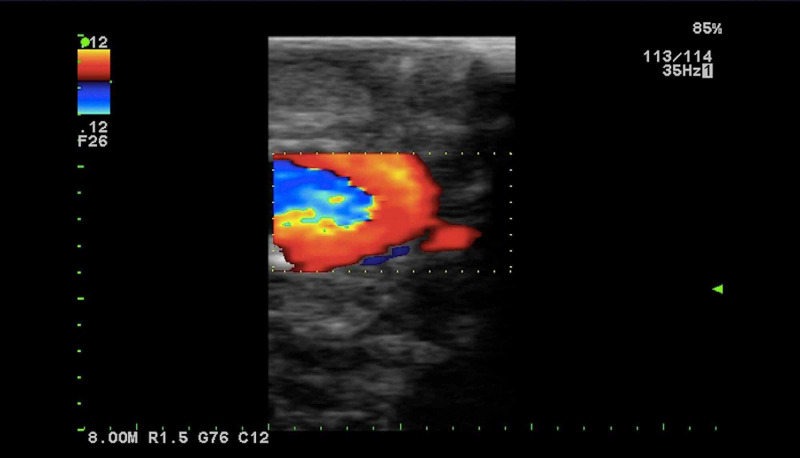
Doppler ultrasonography of the cavernous internal carotid artery

Upon routine re-imaging, a focus of recurrent tumor was identified; this time within the right clivus posterior to the ICA (Figure 1). Again, our minimally invasive skull base team performed an expanded, endoscopic endonasal resection of the recurrent disease using Brain Lab image-guided navigation. Intraoperatively, in the previously operated irradiated skull base, the normal bony architecture of the sella was absent, resulting in the inability to distinguish the anterior genu of the ICA from the adjacent tumor. Using a Prosound Alpha 7 Premier Ultrasound with the Hitachi pituitary transducer (Hitachi, Tokyo, Japan) (Figure 2), the course of the ICA imaged in real time with dynamic flow, allowing for safe, gross total resection of the tumor posterior to the ICA (Figure 3). After an uncomplicated post-operative course, the patient underwent a second course of CyberKnife radiosurgery, and now more than six months post-treatment, her disease is stable clinically and radiologically.

## Discussion

Though expanded endonasal surgery is now a standard approach at leading skull base centers, gross total resection of clival chordomas continues to pose a challenge to skull base surgeons given the close proximity of the brainstem and basilar, vertebral and internal carotid arteries [4]. Landmark-based surgery, neuronavigation, and even acoustic ultrasound are generally adequate to prevent violation of critically important structures during primary surgery. However, in the setting of prior operation, radiation, or extensive, invasive disease, the normal bony architecture of the skull base may be disrupted, and landmarks may be altered or absent. In such cases, there is room for improved technology to guide surgeons. This case demonstrates the potential of real-time endonasal ultrasound for the identification of critical structures along the sella and clivus that drastically altered. This region is particularly vulnerable and amenable to this technology due to the size of the vessels and close arrangement of the carotids, vertebral/basilar system, and brainstem. In our experience, the pituitary transducer can be used on structures facing laterally, but the current tip design makes imaging structures directly in front of the probe a challenge. In combination with neuronavigation, Doppler ultrasonography provides the surgeon with valuable real-time information regarding the location of critical vascular structures. Potential advantages of the endonasal Doppler ultrasound include the ability to measure the distance of the probe in proximity to critical vascular structures, and possibly the ability to measure the amount of residual tumor, though this application has yet to be explored.

## Conclusions

This case demonstrates the feasibility and potential of ultrasonography for use in expanded endonasal surgery of the sella and clivus. In the setting of prior operation, radiation, or extensive disease, the normal bony architecture of the sella may be disrupted, and dynamic imaging is a promising way to overcome this challenge. The feasibility of ultrasound use during the endoscopic endonasal approach has previously been demonstrated. We report what we believe is the first use of intraoperative Doppler ultrasonography during the endoscopic endonasal approach in the setting of a previously operated, and radiated sella.
